# Early-life exposure to indoor air pollution or tobacco smoke and lower respiratory tract illness and wheezing in African infants: a longitudinal birth cohort study

**DOI:** 10.1016/S2542-5196(17)30134-1

**Published:** 2017-11

**Authors:** Aneesa Vanker, Whitney Barnett, Lesley Workman, Polite M Nduru, Peter D Sly, Robert P Gie, Heather J Zar

**Affiliations:** a Department of Paediatrics and Child Health, Red Cross War Memorial Children's Hospital, University of Cape Town, Rondebosch, South Africa; b MRC Unit on Child & Adolescent Health, University of Cape Town, Rondebosch, South Africa; c Children's Health and Environment Program, Child Health Research Centre, The University of Queensland, South Brisbane, QLD, Australia; d Department of Paediatrics and Child Health, Tygerberg Children's Hospital, Stellenbosch University, Tygerberg, South Africa

## Abstract

**Background:**

Indoor air pollution (IAP) and environmental tobacco smoke (ETS) are associated with lower respiratory tract illness (LRTI) or wheezing in children. However, the effect of the timing of these exposures, specifically antenatal versus postnatal, and of alternate fuel sources such as the increasingly used volatile organic compounds have not been well studied. We longitudinally investigated the effect of antenatal or postnatal IAP and ETS on LRTI or wheezing prevalence and severity in African infants.

**Methods:**

Mother and infant pairs enrolled over a 3-year period in a birth cohort study in two centres in Paarl, South Africa, were followed for the first year of life for LRTI or wheezing illness. We measured exposure to IAP (particulate matter, nitrogen dioxide, sulphur dioxide, carbon monoxide, and volatile organic compounds benzene and toluene) using devices placed in homes, antenatally and postnatally. We measured ETS longitudinally by maternal self-report and by urine cotinine measures. Study staff trained in recognition of LRTI or wheeze documented all episodes, which were categorised according to WHO case definition criteria. We used multivariate logistic and Poisson regressions to explore associations.

**Findings:**

Between March 1, 2012, and March 31, 2015, we enrolled 1137 mothers with 1143 livebirths. Of 1065 infants who attended at least one study visit, 524 episodes of LRTI occurred after discharge with a wheezing prevalence of 0·23 (95% CI 0·21–0·26) episodes per child year. Exposures associated with LRTI were antenatal maternal smoking (incidence rate ratio 1·62, 95% CI 1·14–2·30; p=0·004) or particulate matter (1·43, 1·06–1·95; p=0·008). Subanalyses of LRTI requiring hospitalisation (n=137) and supplemental oxygen (n=69) found antenatal toluene significantly increased the risk of LRTI-associated hospitalisation (odds ratio 5·13, 95% CI 1·43–18·36; p=0·012) and need for supplemental oxygen (13·21, 1·96–89·16; p=0·008). Wheezing illness was associated with both antenatal (incidence rate ratio 2·09, 95% CI 1·54–2·84; p<0·0001) and postnatal (1·27, 95% CI 1·03–1·56; p=0·024) maternal smoking. Antenatally, wheezing was associated with maternal passive smoke exposure (1·70, 1·25–2·31; p=0·001) and, postnatally, with any household member smoking (1·55, 1·17 −2·06; p=0·002).

**Interpretation:**

Antenatal exposures were the predominant risk factors associated with LRTI or wheezing illness. Toluene was a novel exposure associated with severe LRTI. Urgent and effective interventions focusing on antenatal environmental factors are required, including smoking cessation programmes targeting women of childbearing age pre-conception and pregnant women.

**Funding:**

Bill & Melinda Gates Foundation, Discovery Foundation, South African Thoracic Society AstraZeneca Respiratory Fellowship, Medical Research Council South Africa, National Research Foundation South Africa, and CIDRI Clinical Fellowship.

## Introduction

Lower respiratory tract illness (LRTI), principally pneumonia, remains the leading cause of under-5 mortality in low-income and middle-income countries (LMICs), with a very high burden of disease in LMIC settings including Africa.^1^ Wheezing illness is common in young children and asthma is the most common non-communicable disease in African children.^2^ Indoor air pollution (IAP) and environmental tobacco smoke (ETS) exposure have been strongly associated with the development of childhood respiratory illness, but little data are available on the effect of the timing of exposures on child respiratory health.^3^, ^4^

Antenatally, in-utero tobacco smoke exposure has been shown to affect lung growth and predispose to development of LRTI or wheezing disorders.^5^ Potential mechanisms include the toxic effects of the numerous chemicals found in tobacco smoke on the developing respiratory system,^6^ suppression of fetal breathing or direct genotoxicity,^7^ the effects of nicotine on lung collagen deposition,^6^ and impaired immune function from imbalances in T-helper-1 and T-helper-2 cell responses.^8^ Although its role is less clear, antenatal IAP exposure is postulated to affect lung development through an interplay of maternal and placenta-fetal factors including oxidative stress resulting in placental insufficiency with decreased transport of oxygen and nutrients to the developing fetus.^9^ Postnatal IAP or ETS exposure might disrupt pulmonary defences leading to epithelial inflammation and affect microbial colonisation and systemic inflammation, particularly if the alveolar capillary membrane is breached.^3^ Most studies have focused on the association of postnatal IAP exposure on child respiratory health;^10^ separating the effects of antenatal versus postnatal exposure is difficult, with few studies able to delineate this.^9^, ^11^

**Research in context**

**Evidence before this study**

Indoor air pollution (IAP) or environmental tobacco smoke (ETS) exposure are important risk factors for childhood lower respiratory tract illness (LRTI) or wheezing, but few data are available on the effect of antenatal compared with postnatal exposures and from low-income or middle-income countries (LMICs) and African settings, which have a high burden of illness. Furthermore, data are scarse on the effect of exposure to new alternate sources of fuel, including volatile organic compounds, increasingly used globally. We searched PubMed, Scopus, and Google Advanced Scholar using the search terms “child”, “indoor air pollution (IAP)”, “tobacco smoke”, “pneumonia”, “respiratory tract infection”, and “wheezing” for articles published in English between Jan 1, 1990, and May 30, 2017. We focused on studies particularly from LMICs that examined the effects of environmental exposures (either IAP or tobacco smoke) with paediatric respiratory health as an outcome. These studies reported an association between environmental exposures and childhood LRTI or wheezing, with IAP associated with almost double the risk of development of LRTI in a systematic review. A similar increase was reported in a systematic review of ETS exposure and LRTI or wheezing, which found that both parental and household smokers significantly increased the risk of LRTI. However, the strongest effect was on bronchiolitis, with household smokers more than doubling this risk.

Highlighting a crucial gap, we found little data differentiating timing of exposures—ie, antenatal versus postnatal exposure—and no longitudinal African data. Furthermore, most studies relied on reported environmental exposures or modelled data, rather than direct measurement of exposures, and none measured new exposures like volatile organic compounds.

**Added value of this study**

In this African birth cohort study, in which exposures were objectively and longitudinally measured antenatally and postnatally, LRTI or wheezing was common and associated with antenatal rather than postnatal exposure to ETS or to IAP. Antenatal exposure to toluene, a volatile organic compound, was identified as a novel exposure associated with LRTI, hospitalisation, and severe disease. Both antenatal and postnatal maternal smoking were associated with wheezing. This study provides novel data on new exposures such as volatile organic compounds that are increasingly used as alternate fuel sources globally. Furthermore, the study highlights the importance of exposures in the antenatal rather than the postnatal period in determining child respiratory health.

**Implications of all the available evidence**

Preventive strategies should focus on women of childbearing age in the prenatal period to reduce ETS and IAP exposure. Alternate sources of fuel might not be as safe as currently regarded; further study of these fuels is needed. Effective public health interventions targeting environmental antenatal and early-life exposures are needed to promote child respiratory health.

Many peri-urban communities, particularly in LMICs including South Africa, are undergoing rapid urbanisation. This development has led to a shift in the type of IAP exposure, with less use of open fires but increasing use of cheap fuels such as paraffin, which produce volatile organic compounds on combustion.^12^ The effect of these on child respiratory health have not been well studied. Furthermore, longitudinal African data are scarse, despite the high incidence of LRTI or wheezing illness,^13^, ^14^ large childhood populations, and exposure to different forms of IAP and ETS.^15^ The prevalence of ETS exposure is also under-reported, particularly in LMICs,^4^ with most studies reporting cross-sectional associations without objective measures of exposure, and in which the extent and effect of exposures on child respiratory health have not been well studied, especially in infants.

The aim of this study was to longitudinally investigate antenatal and postnatal exposure to IAP or ETS, using objective measurements, and the association with LRTI or wheezing illness in a South African birth cohort study.

## Methods

### Study design and participants

We did a longitudinal study of children enrolled in the Drakenstein Child Health Study (DCHS),^16^ a birth cohort study in a peri-urban area of South Africa that included follow-up through the first year of life. Consecutive consenting pregnant women were enrolled at 20–28 weeks' gestation at two public primary health clinics serving different populations: Mbekweni (serving a predominantly black African population) and Newman (serving a predominantly mixed-race population)^16^ from March 1, 2012, to March 31, 2015. We chose a 3-year period for the DCHS study so as to ensure constant enrolment over different seasons and time periods, with more than 90% of the DCHS population attending the public health service (appendix p 2).^16^ We excluded participants who were younger than 18 years, who did not attend study clinics for postnatal care (and thus could not be readily followed up), or who were intending to move out of the district within 2 years after the infant's birth.^16^ All children were born at Paarl Hospital (Paarl, South Africa). Mother and infant pairs were followed at 6–10 weeks, 14 weeks, and 6, 9, and 12 months after birth. Study questionnaires and clinical data were collected at enrolment and at each follow-up visit. We applied a composite socioeconomic status score to each participant and categorised them into quartiles as lowest, low-to-moderate, moderate-to-high, or highest socioeconomic status (appendix p 2).^12^, ^16^, ^17^

The study was approved by the Faculty of Health Sciences Human Research Ethics Committees of the University of Cape Town and of Stellenbosch University, and by the Western Cape Provincial Health Research committee.

### Exposure assessment

An antenatal (within 4 weeks of enrolment) and postnatal (between 4 and 6 months of the infant's life) home visit was undertaken to assess the home environment and measure IAP. Dwellings were categorised^12^ and the most common pollutants and by-products of combustion measured. Particulate matter of diameter 10 μm or less (PM_10_) was measured using a personal air sampling pump (AirChek 52; SKC, Eighty Four, PA, USA) and carbon monoxide with an Altair (Troy, MI, USA) carbon monoxide single gas detection unit, left in homes for 24 h. Diffusion tubes placed in homes for 2 weeks measured nitrogen dioxide, sulphur dioxide (Radiello absorbent filters in polyethylene diffusive body; Sigma-Aldrich, St Louis, MO, USA), and the volatile organic compounds benzene and toluene (Markes thermal desorption tubes; Llantrisant, UK). As described previously,^12^ an average concentration based on the 2-week duration in the home was obtained for nitrogen dioxide, sulphur dioxide, and volatile organic compounds; 24-h averages were obtained for PM_10_. Carbon monoxide data were downloaded to a computer and the frequency of exceedance above the hourly ambient standard was calculated (appendix p 2).^12^ The South African National Ambient Air Quality Standards^18^ were used to define expected exposure levels for each pollutant based on an averaging period of 1 year for each measure: PM_10_ 40 μg/m^3^; nitrogen dioxide 40 μg/m^3^**;** benzene 5 μg/m^3^; toluene 240 μg/m^3^; and carbon monoxide 30 mg/m^3^ (based on an averaging period of 1 h; no more than 88 h of exceedence per year; appendix p 2).^18^ During the postnatal home visit, these same measurements were repeated.

To measure exposure to ETS, questionnaires of maternal and paternal smoking and household exposure to tobacco smoke were administered at enrolment, at the antenatal visit, and at each follow-up visit during the postnatal follow-up period.^19^ Maternal exposure to ETS was also measured using urine cotinine at the second antenatal visit (28–32 weeks' gestation) and at birth, with the highest result used to assign the mother's smoking status (appendix p 2).^19^ Urine cotinine levels were classified as less than 10 ng/mL (non-smoker), 10–499 ng/mL, (passive smoker or exposed), or 500 ng/mL or more (active smoker).^19^

### Assessment of LRTI

We categorised respiratory disease as an episode of LRTI or wheeze. Study staff trained in the recognition of LRTI or wheezing illness documented all episodes, either ambulatory or hospitalised. We defined LRTI and severe LRTI using WHO case definition criteria (appendix p 2).^13^, ^20^ Active surveillance for LRTI in the cohort was established (appendix p 2).^13^ LRTI which occurred at or shortly after birth prior to discharge was defined seperately. Episodes of wheeze were self-reported by a caregiver at a study visit or diagnosed on auscultation by trained study staff at a study visit or intercurrent illness. Study staff were trained in the recognition and auscultation of wheezing; caregivers were also trained in clinical recognition (appendix p 2). Recurrent wheezing was defined as two or more episodes of wheezing.

### Statistical analysis

We used simple descriptive statistics to characterise the study population, summarising continuous data as median (IQR) and categorical data as proportions (95% CI). We used Wilcoxon rank-sum test to compare medians and the χ^2^ test to compare proportions. We used mixed-effects Poisson regression clustered around the infant for multivariate analysis of LRTI incidence and multivariable Poisson regression for wheezing; results are presented as incidence rate ratios (IRRs) and 95% CIs. We used univariate mixed effects logistic regression clustered around the infant to explore associations between demographic, household, and socioeconomic characteristics, indoor air pollutants, and smoke exposure between severe versus non-severe LRTI, hospitalised versus ambulatory, LRTI requiring oxygen versus not requiring oxygen, and wheeze at LRTI versus no wheeze in the subset of infants that had an LRTI; results are presented as odds ratios and 95% CIs. Univariate analysis tested the association between environmental and socioeconomic factors and respiratory disease. (appendix p 2). We included variables that were associated with these outcomes and those of clinical relevance in multivariate (mixed effects) logistic regression models to determine the effect of severity of disease. We used the Wilcoxon signed-rank test to compare differences in the median pollutants measured antenatally to postnatally. We included confounding variables (birthweight, sex, ethnicity [site], socioeconomic status, weight-for-age *Z* score [WAZ],^21^ maternal HIV status, crowding, household characteristics, fossil fuel usage, vaccination status, nutritional status, and feeding in the first 6 months status) that showed an effect in the final analysis models (appendix p 2). All statistical tests were two-sided at α=0·05. We used STATA (version 13.0) for all data analysis.

### Role of the funding source

The sponsors of the study had no role in the study design, data collection, data analysis, data interpretation, or writing of the report. All authors had full access to all the data and had final responsibility for the decision to submit for publication.

## Results

Among the 1137 mothers (median age 25·8 years [IQR 22·0–30·8]) who enrolled with 1143 livebirths (including four sets of twins and one triplet), a total of 4521 visits were completed. 1065 children attended at least one of the study visits between birth and 12 months of age (figure). Attendance varied at each timepoint with a minimum of 778 infants and a maximum of 1030 (figure). 119 (10%) children and 116 (10%) mothers were lost to follow-up before the first full year of follow-up (figure).

**Figure:**
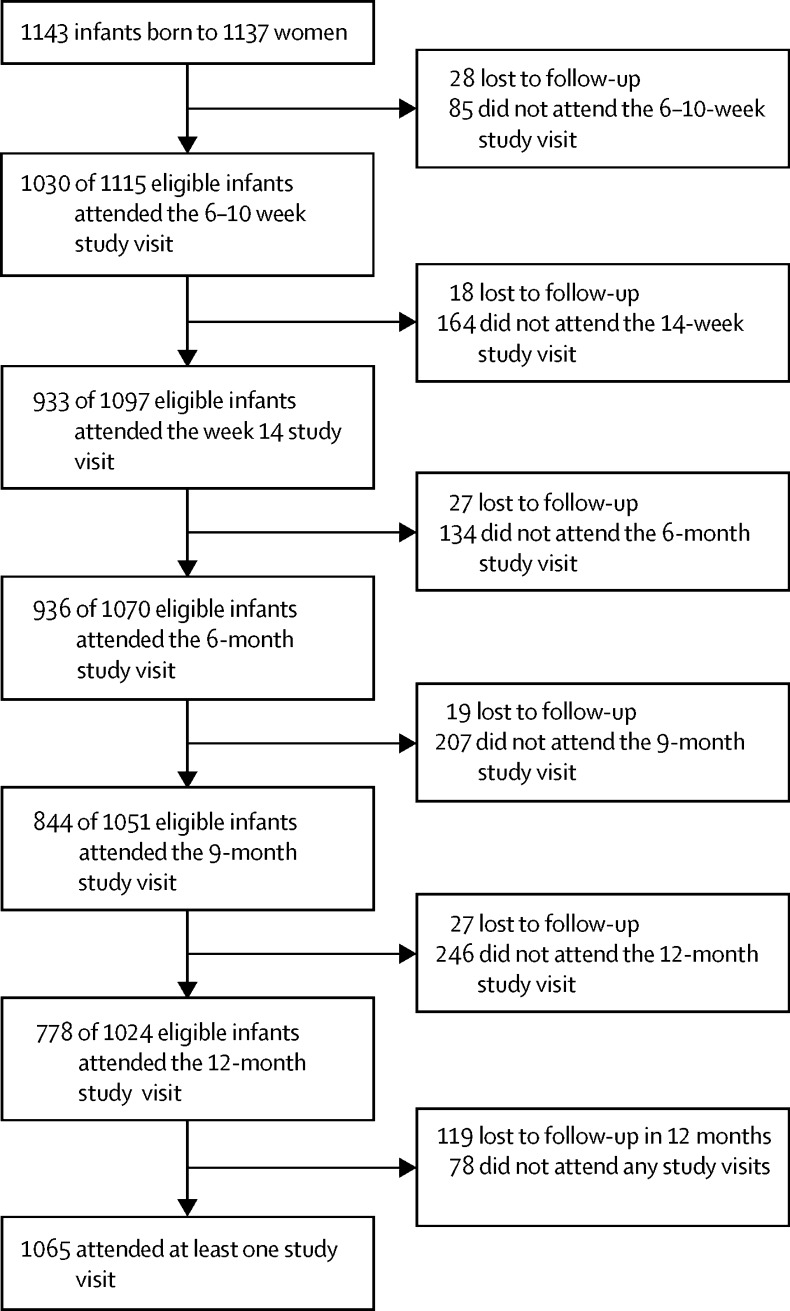
Trial profile The trial profile shows the number of eligible infants assessed at each visit, excluding those who did not attend that specific visit. Eligibility at each visit is defined as all infants minus the total number of infants lost to follow-up by that visit. All infants who attended at least one study visit were assessed, including those lost to follow-up who had attended at least one visit.

We found notable differences between the Mbekweni (black African) and Newman (mixed-race) populations (table 1). More black African participants were in the lowest socioeconomic status quartile than mixed-race participants and the median household size was lower (four people [IQR 3–6] *vs* five people [4–7]) for mixed-race participants (table 1). A third of the 796 homes successfully assessed had fewer than two of the household dimensions; however, 94% of all homes had access to electricity (table 1). Nonetheless, a third of the successfully assessed Mbekweni homes used fossil fuels for cooking and heating (table 1), with paraffin being used in 168 (21%) of 796 homes. 22% of infants were born to HIV-infected mothers and therefore HIV exposed, with a significantly higher proportion of black African infants, but only two infants were HIV infected (table 1). We found no differences between the maternal, household, or birth characteristics of those included in the analysis or those lost to follow-up except in the dwelling catergory, in which 262 (33%) of 796 participants included in the analysis had fewer than two household dimensions compared with 37 (45%) of the 119 participants lost to follow-up (p=0·018).

**Table 1:** Demographic characteristics of the infant cohort and antenatal home environment

			**Mbekweni**	**Newman**	**Total**	**p value**
**Baseline characteristics**						
Number of mothers			583 (55%)	477 (45%)	1060	··
	Age at enrolment, years		26·9 (22·5 to 31·7)	24·8 (21·4 to 29·2)	25·9 (22·1 to 30·8)	<0·0001
Number of infants			588 (55%)	477 (45%)	1065	··
	Male		288 (49%)	260 (55%)	548 (51%)	0·073
	Female		300 (51%)	217 (45%)	517 (49%)	0·073
Preterm*			100 (17%)	75 (16%)	175 (16%)	0·574
Birth WAZ (adjusted for gestation)			−0·41 (−1·22 to 0·24)	−0·73 (−1·36 to −0·06)	−0·54 (−1·31 to 0·09)	<0·0001
HIV exposure			219 (37%)	16 (3%)	235 (22%)	<0·0001
Initiated breastfeeding			430 (73%)	448 (94%)	878 (82%)	<0·0001
Duration of exclusive breastfeeding, months			2·00 (1·00 to 3·65)	2·00 (1·00 to 4·00)	2·00 (1·00 to 4·00)	0·766
Ethnicity						
	Black		581 (99%)	6 (1%)	587 (55%)	<0·0001
	Mixed or other		7 (1%)	471 (99%)	478 (45%)	<0·0001
Socioeconomic status quartiles						<0·0001
	Lowest		176 (30%)	85 (18%)	261 (25%)	··
	Low to moderate		164 (28%)	117 (25%)	281 (26%)	··
	Moderate to high		137 (23%)	134 (28%)	271 (25%)	··
	High		111 (19%)	141 (30%)	252 (24%)	··
**Vaccinations**						
First dose (EPI at 6 weeks)						0·485
	Received on time		484/529 (91%)	404/438 (92%)	888/967 (92%)	··
	Received 2 weeks late		32/529 (6%)	32/438 (7%)	64/967 (7%)	··
Second dose (EPI at 10 weeks)						0·273
	Received on time		438/520 (84%)	368/433 (85%)	806/953 (85%)	··
	Received 2 weeks late		70/520 (13%)	64/433 (15%)	134/953 (14%)	··
Third dose (EPI at 14 weeks)						0·199
	Received on time		510/512 (>99%)	421/422 (>99%)	931/934 (>99%)	··
	Received 2 weeks late		2/512 (<1%)	0	2/934 (<1%)	··
Fourth dose (EPI at 9 months)						0·011
	Received on time		385/471 (82%)	289/380 (76%)	674/851 (79%)	··
	Received 2 weeks late		74/471 (16%)	87/380 (23%)	161/851 (19%)	··
**Home environment**						
Household density						
	Household size		4 (3 to 6)	5 (4 to 7)	4 (3 to 6)	<0·0001
	People per room		2 (1 to 2)	1 (1 to 2)	2 (1 to 2)	0·0036
	People per sleeping room		3 (2 to 4)	3 (2 to 5)	3 (2 to 4)	0·0019
Dwelling category						
	Dimensions†‡					<0·0001
		Two dimensions or fewer	164/421 (39%)	98/375 (26%)	262/796 (33%)	··
		More than two dimensions	257/421 (61%)	277/375 (74%)	534/796 (67%)	··
	Electricity access		535 (91%)	465 (98%)	1000 (94%)	<0·0001
	Fossil fuel (coal, wood, paraffin, or gas) used†					
		Cooking	133/421 (32%)	35/375 (9%)	168/796 (21%)	<0·0001
		Heating	129/421 (31%)	6/375 (2%)	135/796 (17%)	<0·0001

Data are n (%), n/N (%), or median (IQR). WAZ=weight-for-age *Z* score. EPI=Expanded Programme on Immunisation.

* Median gestation for preterm infants in the study was 35 weeks (IQR 32–36).

† Home assessments of dimensions and fossil fuel use were successfully completed for 796 of the 1060 homes.

‡ The six dwelling dimensions were type of home (formal *vs* informal), primary building material (brick or cement *vs* other materials), water supply (piped into dwelling or yard), toilet facilities (non-communal flush), kitchen type (separate room in house), and ventilation in the kitchen area (pipe or duct to exterior).

WAZ differed significantly, with black African babies heavier than mixed-race babies (table 1). Of the 175 (16%) preterm births, most (147 [84%]) were late preterm (>32 weeks). Most mothers initiated breastfeeding, but with a short median duration (table 1). Infant vaccination, including 13-valent pneumococcal conjugate vaccine, was widespread, with more than 80% coverage for the first three doses (table 1).

The median level of each of the pollutants measured did not exceed ambient standards.^18^, ^22^, ^23^ The median PM_10_ level measured antenatally combining both sites was significantly higher than the postnatal measurement (table 2). Of the volatile organic compounds, the median benzene concentration measured antenatally combining both sites was significantly higher than the postnatal measurement (table 2). Use of paraffin for cooking was associated with higher toluene values (p=0·037; table 2). When measures were compared between sites, at both antenatal and postnatal timepoints, only suphur dioxide (antenatally higher in Mbekweni) and carbon monoxide (postnatally higher in Newman) were significantly different (appendix p 3).

**Table 2:** Measured indoor air pollution exposure at antenatal and postnatal home visits

	**Antenatal**	**Postnatal**	**p value**
PM_10_ (μg/m^3^)	33·12 (12·22–64·17)	29·29 (12·59–52·46)	0·011
Nitrogen dioxide (μg/m^3^)	7·08 (3·32–12·70)	5·83 (2·58–12·55)	0·812
Sulphur dioxide (μg/m^3^)	0·00 (0·00–0·28)	0·00 (0·00–0·00)	0·058
Benzene (μg/m^3^)	4·29 (1·70–11·53)	3·12 (1·09–9·46)	0·014
Toluene (μg/m^3^)	16·94 (7·05–44·85)	15·52 (5·93–49·95)	0·869
Average carbon monoxide per h (mg/m^3^)	0·00 (0·00–7·65)	0·00 (0·00–0·00)	0·923

Data are median (IQR) and calculated on the basis of matched pairs (based on Wilcoxon signed-rank test)· PM_10_=particulate matter of diameter 10 μm or less.

Based on antenatal maternal urine cotinine levels, 325 (32%) of 1001 mothers who completed the antenatal assessment were active smokers and 446 (45%) were exposed to tobacco smoke (table 3). Smoking prevalence was significantly higher in mixed-race mothers than in black African mothers (table 3). Self-reported smoking correlated well with urine cotinine measurements, especially in mixed race women (appendix p 3). We found high levels of reported smoke exposure to infants throughout the first year (table 3). In 74% of homes, at least one household member was reported as a smoker (table 3).

**Table 3:** Tobacco smoking and environmental tobacco smoke exposure by study site

			**Mbekweni**	**Newman**	**Total**	**p value**
Antenatal tobacco smoke exposure						<0·0001
	Number of mothers		542	459	1001	
		Urine cotinine <10 ng/mL (non-smoker)	179 (33%)	51 (11%)	230 (23%)	<0·0001
		Urine cotinine 10–499 ng/mL (passive or exposed)	279 (51%)	167 (36%)	446 (45%)	<0·0001
		Urine cotinine ≥500 ng/mL (active smoker)	84 (15%)	241 (53%)	325 (32%)	<0·0001
Self-reported smoking during infancy						<0·0001
	Number of participants (mothers)		583	477	1060	
		Mother	43 (7%)	280 (59%)	323 (30%)	<0·0001
		Father	271 (46%)	320 (67%)	591 (56%)	<0·0001
		Other household members	181 (31%)	358 (75%)	539 (51%)	<0·0001
Total number of household smokers						<0·0001
	Number of participants (mothers)		583	477	1060	
		None	239 (41%)	41 (9%)	280 (26%)	<0·0001
		One	208 (36%)	87 (18%)	295 (28%)	<0·0001
		Two	121 (21%)	176 (37%)	297 (28%)	<0·0001
		Three or more	15 (3%)	173 (36%)	188 (18%)	<0·0001

There were 569 cases of LRTI, of which 45 (8%) occurred at or shortly after birth, before discharge, and were analysed separately. Of 524 LRTI cases occurring after discharge, more occurred among black African infants (321 [61%]) than mixed-race infants (203 [39%]; p<0·0001). The median age at LRTI was 4·6 months (IQR 2·8–7·4). The highest number of cases (178 [37%]) occurred in winter. 105 (20%) of all cases were severe, 137 (26%) required hospitalisation, and 69 (13%) required supplemental oxygen. We observed five (1%) LRTI-related deaths.

The overall prevalence for wheeze per child year was higher among mixed-race infants (0·32, 95% CI 0·27–0·37) than black African infants (0·16, 0·13–0·20; p<0·0001. Recurrent wheeze was uncommon (table 4). Among LRTI cases, 227 (43%) had associated wheeze on auscultation.

**Table 4:** Wheezing in infants at follow-up study visits and cumulative wheeze at 1 year

		**6–10 weeks (n=1030)**	**14 weeks (n=933)**	**6 months (n=936)**	**9 months (n=844)**	**12 months (n=778)**	**Cumulative (n=1065)**
Visit numbers							
	Mbekweni	560	504	503	436	397	588
	Newman	470	429	433	408	381	477
Caregiver-reported wheeze							
	Mbekweni	22/560 (4%)	17/504 (3%)	19/503 (4%)	7/436 (2%)	19/397 (5%)	77/588 (13%)
	Newman	31/470 (7%)	28/429 (7%)	37/433 (9%)	29/408 (7%)	45/381 (12%)	135/477 (28%)
	Total	53 (5%)	45 (5%)	56 (6%)	36 (4%)	64 (8%)	212 (20%)
Treated for wheeze		27/53 (51%)	21/45 (47%)	37/56 (67%)	20/36 (56%)	43/64 (67%)	129/212 (61%)
Prevalence per visit (95% CI)		0·05 (0·03–0·07)	0·05 (0·04–0·06)	0·06 (0·05–0·08)	0·04 (0·03–0·06)	0·08 (0·06–0·10)	0·23 (0·21–0·26)
Recurrent wheeze (≥2 episodes)		4 (<1%%)	14 (2%)	15 (2%)	10 (1%)	6 (1%)	47 (4%)

Antenatal maternal smoking was associated with an increased risk of LRTI, as was male sex (table 5). Increased infant age was associated with a decreased risk of LRTI (table 5). Antenatal PM_10_ above ambient standards (>40 μg/m^3^) was significantly associated with LRTI (table 5). In children with LRTI, antenatal exposure to toluene above ambient standards (>240 μg/m^3^) significantly increased the odds of hospitalisation (odds ratio 5·13, 95% CI 1·43–18·36; p=0·012; appendix pp 4–5) and of requirement for oxygen (13·21, 1·96–89·16; p=0·008; appendix pp 6–7). We found no significant exposures associated with WHO-defined severe LRTI, but the number of severe cases (n=44) meant the model was not sufficiently powered. We also found no associations between antenatal exposures and cases of congenital LRTI.

**Table 5:** Multivariate analysis for lower respiratory tract illness and antenatal environmental exposures

		**Tobacco smoke exposure (n=1059)**		**Indoor air pollutant exposure (n=763)**	
		IRR (95% CI)	p value	IRR (95% CI)	p value
Site: Mbekweni (*vs* Newman)		1·43 (1·07–1·90)	0·009	1·02 (0·76–1·36)	0·872
Maternal smoke status (*vs* non-smoker)					
	Active smoker	1·62 (1·14–2·30)	0·004	··	··
	Passive smoker	1·04 (0·76–1·41)	0·483	··	··
PM_10_ above ambient standard		··	··	1·43 (1·06–1·95)	0·008
Infant characteristics					
	Male	1·69 (1·33–2·13)	<0·0001	1·76 (1·34–2·31)	<0·0001
	WAZ at birth*	0·96 (0·86–1·06)	0·239	0·89 (0·79–1·00)	0·063
	Maternal HIV exposure	1·12 (0·83–1·50)	0·488	1·02 (0·72–1·46)	0·833
	Age, months*	0·90 (0·88–0·92)	<0·0001	0·91 (0·89–0·94)	<0·0001
Socioeconomic status quartiles (*vs* highest)					
	Lowest	1·12 (0·79–1·59)	0·485	1·15 (0·78–1·69)	0·324
	Low to moderate	1·42 (1·02–1·97)	0·042	1·46 (1·01–2·12)	0·039
	Moderate to high	0·98 (0·70–1·39)	0·918	0·99 (0·67–1·47)	0·885

IRR=incidence rate ratio. WAZ=weight-for-age *Z* score. PM_10_=particulate matter of diameter 10 μm or less.

* Per unit increase.

Antenatal maternal smoking increased the risk of infant wheezing, as did passive smoke exposure (table 6). None of the IAP exposures were associated with an increased risk of wheezing (table 6). When correcting for both smoke exposure and IAP, a moderate-to-high socioeconomic status was associated with an increased risk of wheezing (IRR 1·53, 95% CI 1·17–2·00; p=0·002; appendix p 11). Neither postnatal self-reported maternal or household smoking nor PM_10_ exposure was associated with an increased risk of LRTI or of LRTI-associated hospitalisation (appendix pp 4–8).

**Table 6:** Multivariable analysis for infant wheezing and antenatal environmental exposures

		**Tobacco smoke exposure (n=830)**		**Indoor air pollutant exposure (n=585)**	
		IRR (95% CI)	p value	IRR (95% CI)	p value
Maternal smoke status (*vs* non-smoker)					
	Active smoker	2·09 (1·54–2·84)	<0·0001	··	··
	Passive smoker	1·70 (1·25–2·31)	0·001	··	··
Indoor air pollution (*vs* at or below ambient standard)					
	Toluene above ambient standard	··	··	1·29 (0·88–1·89)	0·197
	PM_10_ above ambient standard	··	··	0·93 (0·70–1·25)	0·643
	Benzene above ambient standard	··	··	1·08 (0·85–1·38)	0·539
Infant characteristics					
	Male	1·41 (1·16–1·72)	0·001	1·50 (1·19–1·91)	0·001
	WAZ at birth*	0·98 (0·89–1·07)	0·614	0·95 (0·85–1·06)	0·327
	Maternal HIV exposure	0·49 (0·33–0·72)	<0·0001	0·55 (0·34–0·90)	0·018
Socioeconomic quartiles (*vs* highest)					
	Lowest	0·95 (0·70–1·30)	0·760	0·99 (0·67–1·45)	0·942
	Low to moderate	1·23 (0·93–1·63)	0·151	1·51 (1·07–2·13)	0·019
	Moderate to high	1·51 (1·15–1·98)	0·003	1·62 (1·15–2·27)	0·006
Duration of infant being exclusively breast fed, months*		0·98 (0·93–1·03)	0·435	0·99 (0·93–1·05)	0·740

Site excluded from these analyses as significant confounder. IRR=incidence rate ratio. WAZ=weight-for-age *Z* score. PM_10_=particulate matter of diamater 10 μm or less.

* Per unit increase.

None of the postnatal IAP types measured were associated with wheeze, but postnatal maternal smoking (IRR 1·27, 95% CI 1·03–1·56; p=0·024) and any household member smoking (1·55, 1·17–2·06; p=0·002) were associated with an increased risk of infant wheezing (appendix p 9).

Although combined antenatal and postnatal ETS exposure increased the risk of wheezing (IRR 1·79, 95% CI 1·34–2·38; p<0·0001), this risk was similar to that associated with antenatal exposure alone (appendix p 10). Furthermore, combined ETS and IAP exposure increased the risk of wheezing (1·96, 1·32–2·92; p=0·0001); however, this risk was also similar to that associated with either ETS or IAP exposure alone (appendix p 11). Combined antenatal and postnatal ETS exposure or combined IAP exposure was not associated with a risk of LRTI (appendix pp 10–11).

## Discussion

A high incidence of LRTI or wheezing illness was found in infants in this poor peri-urban community, associated with a very high incidence of exposure to tobacco smoke and IAP despite median measured levels not exceeding acceptable ambient standards. Antenatal exposures were much more strongly associated with respiratory disease in the first year of life, with antenatal maternal smoking, ETS exposure, PM_10_ exposure, or toluene exposure associated with LRTI, wheezing, or hospitalisation for respiratory illness. Among postnatal exposures, only maternal smoking and any household member smoking was associated with an increased risk of wheezing in infants. Recurrent wheezing was unusual, as might be expected in the first year of life.

The effect of antenatal ETS exposure might relate to high levels of in-utero exposure with higher levels than those occurring postnatally. This theory is consistent with our findings in this cohort, in whom infant urine cotinine levels at birth in babies born to mothers who smoke attained levels equivalent to those of an active smoker, but reduced at 6–10 weeks of age to levels indicative of passive exposure associated with maternal smoking.^19^ Furthermore, antenatal exposure might occur at a crucial time of lung development, impairing lung growth.^7^ In-vitro studies have shown that nicotine impairs lung growth and increases collagen deposition in airways.^6^ The very high prevalence of maternal smoking in pregnancy—particularly in the mixed-race population, which was up to ten times higher than the reported African pooled prevalence^24^—and high exposure to tobacco smoke in utero are concerning. The results might not be generalisable to settings with lower levels of smoke exposure; however, maternal smoking prevalence is rising in Africa and among pregnant women.^24^ Furthermore, self-reported smoking is under-reported by pregnant women; however, in our study self-reported smoking and urine cotinine measurements correlated closely, especially in the mixed-race, high-prevalence smoking community.

A few studies^25^, ^26^ have tried to differentiate timing of exposure on the development of childhood respiratory illness with difficulty in measuring the effect of antenatal exposure compared with postnatal exposure. In this study, antenatal exposure was the most important risk associated with the development of respiratory illness in infants.

The differences between antenatal and postnatal measurements of PM_10_ were due to a combination of seasons and sites. Antenatal exposure to PM_10_ was associated with an increased risk of LRTI, as has been previously reported.^10^, ^27^, ^28^ This result might be due to impaired lung growth and increased risk of infection associated with exposure.^29^ Furthermore, innate immune responses might be compromised due to impairment of alveolar macrophage function and upregulation of inflammatory responses.^30^, ^31^, ^32^ Particulate matter inhaled during pregnancy might therefore act directly on the developing fetus or induce a systemic immune or inflammatory response resulting in placental insufficiency leading to reduced fetal oxygen and nutrients.^9^, ^33^ By comparison, postnatal exposure relies on direct inhalation of PM_10_ that results in increased number of macrophages, neutrophils, and T lymphocytes in the lungs.^31^ The antenatal developmental factors increased the susceptibility to LRTI more than postnatal exposure did, particularly in the first months of life.

A novel finding was the association between antenatal toluene exposure and severe LRTI, with exposure increasing the risk of hospitalisation by almost five times and the need for supplemental oxygen more than 13 times. Toluene has numerous sources including ETS, paraffin, solvents, emissions, and household products,^34^ reflective of the sources of IAP in many poor peri-urban communities. Although toluene exposure has been reported to play a part in wheezing illnesses and asthma development or exacerbations, no studies have described the association of antenatal toluene exposure with LRTI in children.^35^, ^36^ Consistent with the findings for other IAP exposures, postnatal exposure was not associated with LRTI incidence or severity. In-vitro studies have shown an effect on immune cells including suppression of cytokine secretion and lymphocyte activity, so potentially increasing susceptibility to severe LRTI.^37^ Furthermore, antenatal maternal exposure to IAP might affect the developing fetal innate immune system—in particular toll-like receptors and nucleotide-binding oligomerisation domain-like receptors involved in pathogen-induced immune responses,^38^ which might contribute to the severity of LRTI, as occurred in infants with antenatal toluene exposure. Mice models have also shown a shift in balance from Th1 and Th2 responses to predominantly Th2 responses with toluene exposure.^39^ Although the small number of severe cases of LRTI might be a limitation of this observation, this association requires further investigation, particularly because volatile organic compound exposures are ubiquitous, increasing globally, and often under-recognised.

The incidence of LRTI and prevalence of wheezing was high, with important differences in the two communities. Although LRTI was more common in black African infants, wheezing was more prevalent in mixed-race infants, even though more than 40% of LRTI was associated with wheezing. The higher prevalence of wheezing in mixed-race infants might be explained by high exposure to ETS from antenatal maternal smoking and household smoking. The higher prevalence of LRTI in black African infants might be explained by their poorer socioeconomic status, with more homes missing basic household dimensions, higher HIV exposure, and associated household exposure to potential pathogens or greater use of fossil fuels for cooking and heating.^12^ We explored the effects of other recognised risk factors associated with LRTI including crowding, nutritional status, and immunisation, but found no significant associations. However, immunisation rates in both communities were high and nutrition was generally good.

Strengths of this study include the longitudinal follow-up, prospective collection of data, high cohort retention, and repeated objective measures of IAP and ETS through the antenatal period and through infancy. Few studies, particularly from LMICs, have directly measured household IAP exposures in large numbers.^40^ The strong association between antenatal exposures and LRTI including severe LRTI, which did not occur with postnatal exposures, suggests that in-utero exposures might be important in determining susceptibility to LRTI in infancy. This result might be mediated through effects on lung function, as substudies of the DCHS have previously shown that antenatal smoke exposure is associated with lower lung function and lower respiratory system compliance in these infants shortly after birth.^41^, ^42^ Limitations of this study include the broad clinical definition of LRTI used. However, the WHO definitions are widely used for maximum sensitivity and to reflect the broad spectrum of LRTI. A further limitation was reliance on caregiver report of wheezing episodes. However, physician-diagnosed wheezing also occurred at follow-up or sick visits and at the time of LRTI. Furthermore, large epidemiological studies such as the International Study of Asthma and Allergies in Childhood^2^ have relied on report of wheeze as a standard method. Other limitations were the use of maternal rather than infant birth urine cotinine measures to assess ETS exposure, given that not all infants had urine collected at birth, and no validated postnatal measures of ETS exposure. However, maternal self-report and urine cotinine levels were highly correlated, as was the sensitivity of self-reported household smokers compared with cotinine results.^19^

Antenatal exposures were the most significant exposures associated with LRTI in infancy, suggesting a developmental lung effect. This study highlights the need for urgent and effective smoking cessation programmes targeting women of childbearing age pre-conception and pregnant women. The study also highlights the importance of other sources of IAP, including toluene exposure, which has not been previously described to be associated with severe LRTI and is increasingly used as rapid urbanisation in LMICs occurs. Limiting of IAP exposure, by identifying household sources of IAP and providing safe alternative fuels, and improving household ventilation^40^, ^43^ could be important strategies to optimise child health. This study underscores the importance of the antenatal period as a time of exposure, by contrast to the postnatal period, which has been the focus of most studies. Further study of this cohort will provide important information on the long-term effects of these exposures on respiratory health in a LMIC population.

## References

[1] Zar HJ, Ferkol TW (2014). The global burden of respiratory disease—impact on child health. Pediatr Pulmonol.

[2] Beasley R, of Asthma TIS (1998). Worldwide variation in prevalence of symptoms of asthma, allergic rhinoconjunctivitis, and atopic eczema: ISAAC. Lancet.

[3] Gordon SB, Bruce NG, Grigg J (2014). Respiratory risks from household air pollution in low and middle income countries. Lancet Respir Med.

[4] Öberg M, Jaakkola MS, Woodward A, Peruga A, Prüss-Ustün A (2011). Worldwide burden of disease from exposure to second-hand smoke: a retrospective analysis of data from 192 countries. Lancet.

[5] Sly PD (2011). The early origins of asthma: who is really at risk?. Curr Opin Allergy Clinl Immunol.

[6] Gibbs K, Collaco JM, McGrath-Morrow SA (2015). Impact of tobacco smoke and nicotine exposure on lung development. Chest.

[7] Grant SG (2010). Tobacco smoke exposure and somatic mutation in newborns. Open Pediatric Med J.

[8] Singh SP, Gundavarapu S, Pena-Philippides JC (2011). Prenatal secondhand cigarette smoke promotes Th2 polarization and impairs goblet cell differentiation and airway mucus formation. J Immuno.

[9] Korten I, Ramsey K, Latzin P (2016). Air pollution during pregnancy and lung development in the child. Paediatr Respir Rev.

[10] Dherani M, Pope D, Mascarenhas M, Smith KR, Weber M, Bruce N (2008). Indoor air pollution from unprocessed solid fuel use and pneumonia risk in children aged under five years: a systematic review and meta-analysis. Bull World Health Organ.

[11] Jones LL, Hashim A, McKeever T, Cook DG, Britton J, Leonardi-Bee J (2011). Parental and household smoking and the increased risk of bronchitis, bronchiolitis and other lower respiratory infections in infancy: systematic review and meta-analysis. Respir Res.

[12] Vanker A, Barnett W, Nduru PM, Gie RP, Sly PD, Zar HJ (2015). Home environment and indoor air pollution exposure in an African birth cohort study. Sci Total Environ.

[13] Zar HJ, Barnett W, Stadler A, Gardner-Lubbe S, Myer L, Nicol MP (2016). Aetiology of childhood pneumonia in a well vaccinated South African birth cohort: a nested case-control study of the Drakenstein Child Health Study. Lancet Respir Med.

[14] Lai CK, Beasley R, Crane J (2009). Global variation in the prevalence and severity of asthma symptoms: phase three of the International Study of Asthma and Allergies in Childhood (ISAAC). Thorax.

[15] Gall ET, Carter EM, Earnest CM, Stephens B (2013). Indoor air pollution in developing countries: research and implementation needs for improvements in global public health. Am J Public Health.

[16] Zar HJ, Barnett W, Myer L, Stein DJ, Nicol MP (2015). Investigating the early-life determinants of illness in Africa: the Drakenstein Child Health Study. Thorax.

[17] le Roux DM, Myer L, Nicol MP, Zar HJ (2015). Incidence and severity of childhood pneumonia in the first year of life in a South African birth cohort: the Drakenstein Child Health Study. Lancet Glob Health.

[18] Government Gazette, Republic of South Africa (Dec 24, 2009). Department of Environmental Affairs. National Ambient Air Quality Standards. https://www.environment.gov.za/sites/default/files/legislations/nemaqa_airquality_g32816gon1210.pdf.

[19] Vanker A, Barnett W, Brittain K (2016). Antenatal and early life tobacco smoke exposure in an African birth cohort study. Int J Tuberc Lung Dis.

[20] WHO (2014). Integrated management of childhood illness: distance learning course.

[21] Fenton TR, Nasser R, Eliasziw M, Kim JH, Bilan D, Sauve R (2013). Validating the weight gain of preterm infants between the reference growth curve of the fetus and the term infant. BMC Pediatr.

[22] Richardson M, Lukey P, Phahlane A, Department of Environmental Affairs, Republic of South Africa State of Air Report 2005. Chapter 3: air quality standards and objectives. https://www.environment.gov.za/sites/default/files/docs/stateofair_executive_iaiquality_standardsonjectives.pdf.

[23] WHO (1989). Indoor air quality: organic pollutants: report on a WHO meeting, Berlin, West, 23–27 August 1987.

[24] Caleyachetty R, Tait CA, Kengne AP, Corvalan C, Uauy R, Echouffo-Tcheugui JB (2014). Tobacco use in pregnant women: analysis of data from Demographic and Health Surveys from 54 low-income and middle-income countries. Lancet Glob Health.

[25] Silvestri M, Franchi S, Pistorio A, Petecchia L, Rusconi F (2015). Smoke exposure, wheezing, and asthma development: A systematic review and meta-analysis in unselected birth cohorts. Pediatr Pulmonol.

[26] den Dekker HT, Sonnenschein-van der Voort AMM, de Jongste JC (2015). Tobacco smoke exposure, airway resistance, and asthma in school-age children: the generation R study. Chest.

[27] Voynow JA, Auten R (2015). Environmental pollution and the developing lung. Clin Pulm Med.

[28] Jedrychowski WA, Perera FP, Spengler JD (2013). Intrauterine exposure to fine particulate matter as a risk factor for increased susceptibility to acute broncho-pulmonary infections in early childhood. Int J Hyg Environ Health.

[29] Grigg J (2009). Particulate matter exposure in children: relevance to chronic obstructive pulmonary disease. Proc Am Thorac Soc.

[30] Brugha R, Grigg J (2014). Urban air pollution and respiratory infections. Paediatr Respir Rev.

[31] Salvi S, Holgate ST (1999). Mechanisms of particulate matter toxicity. Clin Exp Allergy.

[32] Goldizen FC, Sly PD, Knibbs LD (2016). Respiratory effects of air pollution on children. Pediatr Pulmonol.

[33] Kannan S, Misra DP, Dvonch JT, Krishnakumar A (2007). Exposures to airborne particulate matter and adverse perinatal outcomes: a biologically plausible mechanistic framework for exploring potential. Cien Saude Colet.

[34] Chin JY, Godwin C, Parker E (2014). Levels and sources of volatile organic compounds in homes of children with asthma. Indoor Air.

[35] Mendell MJ (2007). Indoor residential chemical emissions as risk factors for respiratory and allergic effects in children: a review. Indoor Air.

[36] Wichmann FA, Muller A, Busi LE (2009). Increased asthma and respiratory symptoms in children exposed to petrochemical pollution. J Allergy Clin Immunol.

[37] Wichmann G, Muhlenberg J, Fischader G (2005). An experimental model for the determination of immunomodulating effects by volatile compounds. Toxicol In Vitro.

[38] Bauer RN, Diaz-Sanchez D, Jaspers I. Effects of air pollutants on innate immunity: the role of Toll-like receptors and nucleotide-binding oligomerization domain-like receptors. *J Allergy Clin Immunol*; **129:** 14–24. 10.1016/j.jaci.2011.11.004 PMC4341993 22196521

[39] Yamamoto S, Tin Tin Win S, Yoshida Y, Kunugita N, Arashidani K, Fujimaki H (2009). Children's immunology, what can we learn from animal studies (2): modulation of systemic Th1/Th2 immune response in infant mice after prenatal exposure to low-level toluene and toll-like receptor (TLR) 2 ligand. J Toxicol Sci.

[40] Gordon SB, Bruce NG, Grigg J (2014). Respiratory risks from household air pollution in low and middle income countries. Lancet Respir Med.

[41] Gray D, Czovek D, Smith E (2015). Respiratory impedance in healthy unsedated South African infants: effects of maternal smoking. Respirology.

[42] Gray D, Willemse L, Visagie A (2016). Determinants of early-life lung function in African infants. Thorax.

[43] Mortimer K, Ndamala CB, Naunje AW (2017). A cleaner burning biomass-fuelled cookstove intervention to prevent pneumonia in children under 5 years old in rural Malawi (the Cooking and Pneumonia Study): a cluster randomised controlled trial. Lancet.

