# Supplementary material for: Early-life exposure to indoor air pollution or tobacco smoke and lower respiratory tract illness and wheezing in African infants: a longitudinal birth cohort study
Source: Lancet Planet Health. 2017 Nov;1(8):e328–36. doi: 10.1016/S2542-5196(17)30134-1 (PMC5681433; doi:10.1016/S2542-5196(17)30134-1)
Supplement: Supplementary appendix [file mmc1.pdf]

THE LANCET
Planetary Health

Supplementary appendix

This appendix formed part of the original submission and has been peer reviewed. We post it as supplied by the authors.

Supplement to: Vanker A, Barnett W, Workman L, et al. Early-life exposure to indoor air pollution or tobacco smoke and lower respiratory tract illness and wheezing in African infants: a longitudinal birth cohort study. *Lancet Planet Health* 2017; **1:** e328–36.

Supplemental information

Early-Life Exposure to Indoor Air Pollution or Tobacco Smoke and Lower Respiratory Illness in African Infants.

Aneesa Vanker[1], Whitney Barnett[1], Lesley Workman[1], Polite M. Nduru[1], Peter D. Sly[2], Robert P. Gie[3], Heather J. Zar[1]

[1] Department of Paediatrics and Child Health, Red Cross War Memorial Children's Hospital, and MRC Unit on Child & Adolescent Health, University of Cape Town, Klipfontein Road, Rondebosch, 7700, South Africa

[2] Children's Health and Environment Program, Child Health Research Centre, The University of Queensland 62 Graham St South Brisbane, Queensland, Australia, 4101

[3]Department of Paediatrics and Child Health, Tygerberg Children's Hospital, Stellenbosch University, Francie van Zijl Avenue, Tygerberg, , 7505, South Africa

Supplemental Table 1: Methods

**Study population and procedures**	
1.1 Health Facilities	Mbekweni and Newman health facilities provide free primary health to women and children, including antenatal care, a strong prevention of mother to child transmission (PMTCT) HIV program, childhood immunizations including thirteen valent pneumococcal conjugate vaccine (PCV13) and care for intercurrent illness. Hospital referral is to the single public hospital serving the area, Paarl hospital.
1.2 Sociodemographic	Sociodemographic data were collected using a questionnaire adapted from the South African Stress and Health Study (SASH).(1) A composite SES score was developed based on current employment status and standardised scores of educational level, household income and a composite asset index made up of access to household resources, amenities and market access categorising participants as being lowest SES, low-moderate SES, moderate-high SES or high SES.
**Measuring exposure to IAP**	
1.3 Dwelling categorization	Dwellings were catergorized as a poor structure if there were 2 or less of 6 dwelling dimensions (type of home, building material, water supply, type of toilet, kitchen type and ventilation).(2) An implementation of the Alkire-Foster method, a flexible technique used to incorporate a number of dimensions of poverty or well-being, that can complement poverty assessment (2, 3) was applied to the dwelling characteristics. Six dwelling factors were used; type of home (formal versus informal), primary building material (brick or cement versus other materials), water supply (piped into dwelling or yard), toilet facilities (non communal flush), kitchen type (separate room in house) and ventilation in the kitchen area (pipe or duct to exterior). Dwellings were then categorised according to the number of dimensions lacking. This method defines a dwelling as a "poor structure" if it lacks one-third or more of the factors considered.(4)
1.4 Pollutant measurement equipment	• Particulate matter (PM_{10}) (personal air sampling pump – SKC Aircheck 52^R)<br>• Carbon monoxide (CO) (Altair^R Carbon Monoxide single gas detection unit)<br>• Nitrogen dioxide (NO) and sulphur dioxide (Radiello^R adsorbent filters in polyethylene diffusive body)<br>• Volatile organic compounds (VOC) benzene and toluene (Markes^R thermal desorption tubes using passive diffusion tubes).(4)<br>All measurements were done in the communal/main living room, away from windows and doors, approximately 1.5 meters from the ground.
1.5 National Ambient Air Quality Standards	Expected exposure for each pollutant based on an averaging period of 1 year for each measure; PM10: 40ug/m^3, NO2: 40ug/m^3, benzene: 5ug/m^3, toluene: 240ug/m^3, CO: >30mg/m^3 (not more than 88 hours). (5) An average concentration based on the 2-week duration in the home was obtained for sulphur dioxide/nitrogen dioxide and volatile organic compounds; 24-hour averages were obtained for particulate matter. Carbon monoxide data was downloaded to a computer and the frequency of exceedance above the hourly ambient standard was calculated. Based on the 10 minute readings, total hourly concentrations were computed using the trapezium rule. Two consecutive ten minute CO readings were used to represent parallel sides of a trapezium and the 10 minute interval to represent the distance between the parallel sides (width). The trapezium formula; half the sum of the parallel sides multiplied by the width, was then applied. The sum of six consecutive trapezia areas to represent total CO concentration in an hour was then calculated. Using this approach hourly concentrations were then determined for the entire duration of the CO device in the household. (4)
**Measuring exposure to ETS**	
1.6 Self-reported exposure	Maternal tobacco smoking and exposure were assessed using detailed self report questionnaires at enrolment. Post-natal follow-up questionnaires on child respiratory health included questions on tobacco smoke exposure from partners and household members. Maternal smoking was quantified as pack years, where one pack year was defined as 20 cigarettes smoked daily for one year. Maternal nicotine dependence was assessed using the Fagerström test for nicotine dependence, a well-validated questionnaire which scores tobacco dependence as low, low to moderate, moderate or high. The Alcohol, Smoking and Substance Involvement Screening Test (ASSIST) was administered to assess substance use and substance-related risk.(6)
1.7 Urine cotinine measurement	Urine cotinine tests were performed using the IMMULITE^R 1000 Nicotine Metabolite Kit (Siemens Medical Solutions Diagnostics^R, Glyn Rhonwy, United Kingdom).(7)
**Assessing lower respiratory disease**	
1.8 WHO pneumonia/LRTI case definitions	WHO pneumonia/LRTI case definition: cough or difficulty breathing and age-specific tachypnea or lower chest wall in-drawing).(8)<br>WHO severe pneumonia/LRTI case definition: any child under 2 months of age with signs of pneumonia/LRTI or in a child of any age with danger signs (cyanosed, unable to drink, seizures, or decreased level of consciousness).(8, 9)
1.9 Surveillance for pneumonia/LRTI	Active surveillance for pneumonia/LRTI in the cohort was undertaken as described, using community field workers, a short message system (SMS) phone system, ongoing monitoring of cases at health facilities and study staff who could be contacted by a mother at all times.(10)

1.10 Recognition of LRTI/wheezing	Child caregiver reports at each study visit and episodes identified through the active surveillance for respiratory symptoms associated with LRTI was used to measure wheeze. Study nurses at the primary clinics performed active surveillance and assessed presenting infants in real time. (10, 11) Training of nursing staff included video-clips demonstrating clinical signs. The study doctor provided regular on-site refresher training and competency assessment.(10)
**Statistical analysis**	
1.11 Confounding variables	Potential confounding variables included birth weight, gender, ethnicity (site), SES status, weight for age Z (WAZ) score,(12) maternal HIV status, crowding, household characteristics, fossil fuel usage, vaccination status, nutritional status and feeding in the first 6 months status.

Supplemental Table 2: Indoor air pollution (IAP) measurements recorded at antenatal and postnatal visits

	**Antenatal**				**Postnatal**			
**IAP Measure Ambient Concentration**	**Mbekweni Median (IQR)**	**Newman Median (IQR)**	**All Median (IQR)**	**P Value**	**Mbekweni Median (IQR)**	**Newman Median (IQR)**	**All Median (IQR)**	**P Value**
Particulate Matter (PM10) **(ug/m^3)**	31·77 (12·36 - 62·73)	36.04 (13.01 - 65·84)	33·41 (12·49 - 64·80)	0·348	30·29 (14·67 – 51·05)	28·44 (10·47 - 53·71)	29·47 (12·59 - 52·48)	0·328
Nitrogen Dioxide **(ug/m^3)**	6·87 (2·50 - 14·56)	7·12 (3·84 - 11·28)	7·03 (3·31 - 12·66)	0·622	6·34 (2·81 - 14·57)	5·28 (2·48 - 11·25)	5·83 (2·58 - 12·55)	0·130
Sulphur Dioxide **(ug/m^3)**	0·00 (0·00 - 0·34)	0·00 (0·00 - 0·17)	0·00 (0·00 - 0·28)	0·039	0·00 (0·00 - 0·00)	0·00 (0·00 - 0·00)	0·00 (0·00 - 0·00)	0·794
Benzene **(ug/m^3)**	4·50 (1·46 - 17·71)	3·88 (1·83 - 8·56)	4·28 (1·74 - 11·39)	0·637	2·81 (0·75 - 14·41)	3·22 (1·46 - 7·57)	3·08 (1·06 - 9·46)	0·312
Toluene**(ug/m^3)**	16·06 (5·84 - 42·92)	17·54 (8·24 - 46·48)	16·88 (7·04 - 44·57)	0·213	14·72 (4·79 - 48·77)	15·89 (6·52 - 51·66)	15·50 (5·90 - 48·97)	0·286
Average Carbon Monoxide Per Hour **(mg/m^3)**	0·00 (0·00 - 3·22)	0·00 (0·00 - 9·09)	0·00 (0·00 - 6·21)	0·105	0·00 (0·00 - 0·00)	0·00 (0·00 - 5·57)	0·00 (0·00 - 0·00)	0·015

IAP, indoor air pollution; IQR, inter-quartile range

Supplemental Table 3: Correlation of maternal antenatal cotinine and self-reported total smoke exposure

	**Mbekweni**		**Newman**		**All**	
	**Sensitivity**	**Specificity**	**Sensitivity**	**Specificity**	**Sensitivity**	**Specificity**
Estimate, %	71.30	59.00	95.40	35.70	83.60	53.80
95% Confidence Interval, %	66.80 – 75.50	51.70 – 66.00	93.00 – 97.10	23.40 – 49.60	81.00 – 86.00	47.40 – 60.10

Supplemental Table 4: Risk factors for lower respiratory tract illness (LRTI) requiring hospitalization

	**Antenatal Risk Factors**						**Postnatal Risk Factors**					
	**All Episodes N = 524**		**Included in Model N = 245**				**All Episodes N = 524**		**Included in Model N = 127**			
	Amb., N = 387	Hsp., N =137	Amb., N = 177	Hsp., N = 68	*OR (95%CI)*	*P value*	Amb., N = 387	Hsp., N =137	Amb., N = 100	Hsp, N = 27	*OR (95%CI)*	*P value*
**Site**												
Mbekweni	251 (65%)	73 (53%)	82 (46%)	27 (40%)	0·77 (0·35 - 1·72)	0.524	251 (65%)	73 (53%)	50 (50%)	12 (44%)	0·40 (0·10 - 1·66)	0206
**Smoke exposure status**												
Antenatal maternal non-smoker	75 (19%)	22 (16%)	36 (20%)	14 (20%)	1							
Antenatal / Postnatal maternal active smoker	134 (35%)	61 (45%)	70 (40%)	31 (46%)	0·67 (0·28 - 1·60)	0·362	109 (28%)	50 (37%)	33 (33%)	12 (44%)	1·12 (0·23 - 5·45)	0·893
Antenatal maternal passive smoker / Postnatal household smoker	158 (41%)	44 (32%)	71 (40%)	23 (34%)	0·58 (0·23 - 1·50)	0·264	269 (70%)	101 (74%)	77 (77%)	21 (78%)	0·50 (0·12 - 2·19)	0·359
Unknown	20 (5%)	10 (7%)										
**Infant characteristics**												
Gender (male)	240 (62%)	91 (66%)	123 (69%)	50 (74%)	1·05 (0·52 - 2·11)	0·887	240 (62%)	91 (66%)	64 (64%)	20 (74%)	2·10 (0·64 - 6·97)	0·223
WAZ at birth	-0·7 (-1·4, -0·1)	-0·8 (-1·5, 0·0)	-0·8 (-1·5, -0·1)	-0·9 (-1·6, 0·0)	0·64 (0·51 - 0·82)	<0·001	0·2 (-0·6, 1·1)	-0·4 (-1·7, 0·8)	0·6 (-0·3, 1·3)	0.0 (-0·6, 0·9)	1·06 (0·60 - 1·89)	0·836
Maternal HIV exposure	107 (28%)	39 (28%)	22 (12%)	13 (19%)	2·04 (0·76 - 5·45)	0·156	107 (28%)	39 (28%)	9 (9%)	7 (26%)	11·14 (1·71 - 72·73)	0·012
Age (mid-interval in days)	4·7 (3 - 7)	2·5 (1·5 - 7)	5·2 (3·3 - 7·1)	2·9 (1·5 - 8·1)	0·90 (0·82 - 1·00)	0·050	4·7 (3 - 7)	2·5 (1·5 - 7)	5·2 (3·3 - 7·1)	2·3 (1·5 - 8·4)	0·89 (0·74 - 1·07)	0·215
**SES quartiles (compared to high SES)**												
Lowest SES	96 (25%)	37 (27%)	47 (27%)	15 (22%)	0·56 (0·22 - 1·42)	0·221	96 (25%)	37 (27%)	35 (35%)	10 (37%)	0·75 (0·21 - 2·65)	0·659
Low-mod SES	139 (36%)	40 (29%)	64 (36%)	23 (34%)	0·99 (0·41 - 2·37)	0·977	61 (16%)	19 (14%)	17 (17%)	5 (19%)	0·60 (0·14 - 2·53)	0·490
Mod-high SES	83 (21%)	31 (23%)	36 (20%)	12 (18%)	0·75 (0·28 - 1·97)	0·558	74 (19%)	20 (14%)	25 (25%)	3 (11%)	0·27 (0·05 - 1·34)	0·109
High SES	69 (18%)	29 (21%)	30 (17%)	18 (26%)	1		59 (15%)	24 (18%)	23 (23%)	9 (33%)		
Unknown							97 (25%)	37 (27%)				

**Method of feeding**												
Duration of exclusive breast feeding (months)	2.0 (1.0 - 3·4)	2.0 (1.0 - 4.0)	2.0 (1.0 - 3·2)	1·7 (1.0 - 4.0)	0.95 (0·79 - 1·14)	0·577	2.0 (1.0 - 3·4)	2.0 (1.0 - 4.0)	1·9 (1.0 - 5.0)	1·5 (1.0 - 3.0)	0·86 (0·64 - 1·15)	0·304
**Toluene indoor air pollution**												
Below ambient standard	223 (58%)	76 (55%)	172 (97%)	61 (90%)	1		125 (32%)	41 (30%)	88 (88%)	22 (81%)		
Above ambient standard	8 (2%)	7 (5%)	5 (3%)	7 (10%)	5·13 (1·43 - 18·36)	0·012	17 (4%)	5 (4%)	12 (12%)	5 (19%)	1·63 (0·40 - 6·70)	0·500
Unknown	156 (40%)	54 (40%)					245 (64%)	91 (66%)				

WAZ, weight-for-age z-score; HIV, human immunodeficiency virus; SES, socio-economic status; Amb., ambulatory; Hsp., hospitalized

Supplemental Table 5: Risk factors for lower respiratory tract illness (LRTI) requiring oxygen

	Antenatal Risk Factors						Postnatal Risk Factors					
	All Episodes N = 524		Included in Model N = 244				All Episodes N = 521		Included in Model N = 127			
	No Oxygen N = 452	Oxygen N = 69	No Oxygen N = 209	Oxygen N = 35	OR (95%CI)	P value	No Oxygen N = 452	Oxygen N = 69	No Oxygen N = 116	Oxygen N = 11	OR (95%CI)	P value
**Site**												
Mbekweni	285 (63%)	36 (52%)	90 (43%)	18 (51%)	1·57 (0·49 - 5.00)	0·446	285 (63%)	36 (52%)	58 (50%)	4 (36%)	0·06 (0·0 - 21·10)	0·346
**Smoke exposure status**												
Antenatal maternal non-smoker	85 (19%)	10 (14%)	43 (21%)	7 (20%)	1·0							
Antenatal passive smoker (cotinine)	178 (39%)	23 (33%)	78 (37%)	15 (43%)	1·34 (0·36 - 4·92)	0·659						
Antenatal active smoker (cotinine)	166 (37%)	29 (42%)	88 (42%)	13 (37%)	1·03 (0·25 - 4·34)	0·966						
Postnatal maternal smoker							136 (30%)	23 (33%)	41 (35%)	4 (36%)	1·78 (0·03 - 96·25)	0·778
Postnatal household smoker							319 (71%)	48 (70%)	90 (78%)	8 (73%)	0·28 (0·00 - 31·15)	0·597
Unknown	23 (5%)	7 (10%)										
**Infant characteristics**												
Gender (male)	284 (63%)	44 (64%)	149 (71%)	23 (66%)	0·72 (0·26 - 1.98)	0·521	284 (63%)	44 (64%)	75 (65%)	9 (82%)	2·66 (0·06 - 119·69)	0·614
WAZ at birth	-0·8 (-1·4, -0·1)	-0·5 (-1·4, -0·1)	-0·8 (-1·5, -0·1)	-0·8 (-1·6, 0·1)	0·96 (0·62 - 1·51)	0·875	0·2 (-0·7, 1·1)	-0·25 (-1·77, 0·73)	0·6 (-0·5, 1·3)	0·2 (0·0, 0·9)	1·12 (0·22 - 5·85)	0·892
Maternal HIV exposure	127 (28%)	17 (25%)	28 (13%)	6 (17%)	1·54 (0·35 - 6·79)	0·568	127 (28%)	17 (25%)	13 (11%)	3 (27%)	308·86 (0·02 - 5853189)	0·254
Age (mid-interval in days)	4·7 (2·9 - 7·1)	2·3 (1·1 - 4·2)	5·2 (2·9 - 7·4)	2·5 (1·5 - 3·4)	0·76 (0·62 - 0·93)	0·006	4·7 (2·9 - 7·1)	2·35 (1·13 - 4·19)	5·2 (3·3 - 7·5)	2·0 (0·7 - 2·8)	0·48 (0·18 - 1·28)	0·142
**SES quartiles (compared to high SES)**												
Lowest SES	121 (27%)	12 (17%)	58 (28%)	4 (11%)	0·33 (0·07 - 1·59)	0·168	117 (26%)	16 (23%)	43 (37%)	2 (18%)	1·20 (0·01, 100·91)	0·935
Low-mod SES	154 (34%)	24 (35%)	74 (35%)	12 (34%)	1·00 (0·27 - 3·71)	0·994	65 (14%)	15 (22%)	18 (16%)	4 (36%)	6·16 (0·05, 699·62)	0·451
Mod-high SES	94 (21%)	19 (28%)	38 (18%)	10 (29%)	1·35 (0·33 - 5·58)	0·677	81 (18%)	12 (17%)	25 (22%)	3 (27%)	2·45 (0·09, 155·99)	0·672
High SES	83 (18%)	14 (20%)	39 (19%)	9 (26%)	1		74 (16%)	9 (13%)	30 (26%)	2 (18%)		

Unknown							115 (25%)	17 (25%)				
**Method of feeding**												
Duration of exclusive breast feeding (months)	2·0 (1·0 - 3·5)	2·1 (1·0 - 3·6)	1·8 (1·0 -3·2)	2·0 (1·0 – 4·0)	1·10 (0·84 - 1·45)	0·470	4·0 (2·4, 9.0)	3·64 (3·00, 9·14)	4·0 (2·0, 9·1)	3·0 (3·0, 8·9)	0·51 (0·13, 2·01)	0·337
**Toluene indoor air pollution**												
Below ambient standard	263 (58%)	34 (49%)	203 (97%)	29 (83%)	1							
Above ambient standard	9 (2%)	6 (9%)	6 (3%)	6 (17%)	13·21 (1·96 - 89·16)	0·008	22 (5%)	0 (0%)	17 (15%)	0 (0%)	1·00 (0·98, 1·01)	0·514
Unknown	180 (40%)	29 (42%)										

WAZ, weight-for-age z-score; HIV, human immunodeficiency virus; SES, socio-economic status

Supplemental Table 6: Multivariate analysis for lower respiratory tract infection (LRTI) and postnatal environmental exposures

**Smoke Exposure**<br>(N = 875)		
	IRR (95% CI)	P value
Site		
Mbekweni	1·23 (0·89 - 1·70)	0·207
Smoke exposure		
Maternal self-report smoking	1·22 (0·89 - 1·70)	0·221
Any household member self-report smoking	0·94 (0·68 - 1·30)	0·712
Infant characteristics		
Male	1·67 (1·30 - 2·15)	0.000
WAZ at birth	0·93 (0·83 - 1·04)	0·226
Maternal HIV exposure	1·31 (0·90 - 1·91)	0·160
Age in months	0·90 (0·88 - 0·93)	0·000
SES quartiles		
Lowest SES	1·26 (0·87 - 1·83)	0·218
Low-mod SES	1·63 (1·15 - 2·33)	0·006
Mod-high SES	0·98 (0·68 - 1·43)	0·925
High SES	1	
Method of feeding		
Duration of exclusive breast feeding (months)	0·97 (0·91 - 1·04)	0·361
**Indoor Air Pollutant Exposure**<br>(N = 429)		
	IRR (95% CI)	P value
Site		
Mbekweni	0·91 (0·61 - 1·37)	0·661
Indoor air pollutant exposure		
PM10 above ambient standard	0·61 (0·33 - 1·12)	0·110
Infant characteristics		
Male	1·73 (1·18 - 2·52)	0·005
WAZ at birth	0·94 (0·79 - 1·13)	0·523
Maternal HIV exposure	1·43 (0·76 - 2·70)	0·226
Age in months	0·94 (0·90 - 0·98)	0·002
SES Quartiles		
Lowest SES	1·24 (0·73 - 2·11)	0·427
Low-mod SES	1·35 (0·82 - 2·24)	0·240
Mod-high SES	0·78 (0·45 - 1·36)	0·383
High SES	1	
Method of feeding		

Duration of exclusive breast feeding (months)	0·93 (0·85 - 1·02)	0·108

WAZ, weight-for-age z-score; HIV, human immunodeficiency virus; SES, socio-economic status; PM10, particulate matter

Supplemental Table 7: Multivariable analysis for wheezing and postnatal environmental exposures

**Smoke Exposure** (N = 875)		
	IRR (95% CI)	P value
Self-reported smoke exposure		
Maternal smoking	1·27 (1·03 - 1·56)	0·024
Any household member smoking	1·55 (1·17 - 2·06)	0·002
Infant characteristics		
Male	1·44 (1·18 - 1·74)	<0·001
WAZ at birth	0·96 (0·88 - 1·04)	0·334
Maternal HIV exposure	0·58 (0·40 - 0·85)	0·006
SES quartiles		
Lowest SES	0·99 (0·73 - 1·35)	0·973
Low-mod SES	1·28 (0·97 - 1·70)	0·079
Mod-high SES	1·52 (1·16 - 1·99)	0·002
Infant feeding		
Duration exclusively breast fed in months	0·98 (0·93 - 1·03)	0·410
**Indoor Air Pollutant Exposure** (N = 336)		
	IRR (95% CI)	P value
Indoor air pollution		
Toluene above ambient standard	0·60 (0·35 - 1·05)	0·071
Particulate Matter (PM10) above ambient standard	0·84 (0·56 - 1·26)	0·402
Benzene above ambient standard	1·17 (0·87 - 1·57)	0·291
Infant characteristics		
Male	1·45 (1·09 - 1·93)	0·011
WAZ at birth	0·91 (0·80 - 1·04)	0·183
Maternal HIV exposure	0·98 (0·58 - 1·65)	0·934
SES quartiles		
Lowest SES	0·90 (0·57 - 1·42)	0·646
Low-mod SES	1·07 (0·70 - 1·64)	0·758
Mod-high SES	1·78 (1·20 - 2·64)	0·004
Infant feeding		
Duration exclusively breast fed in months	0·89 (0·83 - 0·96)	0·003

WAZ, weight-for-age z-score; HIV, human immunodeficiency virus; SES, socio-economic status

Supplemental Table 8: Multivariate analysis for combined antenatal and postnatal environmental exposures

**Wheeze**		
	IRR (95% CI)	P value
Combined		
Smoke exposure	1·79 (1·34 - 2·38)	<0·001
Infant characteristics		
Male	1·45 (1·20 - 1·77)	<0·001
WAZ at birth	0·95 (0·87 - 1·04)	0·239
Maternal HIV exposure	0·50 (0·34 - 0·74)	0·001
SES quartiles		
Lowest SES	0·94 (0·70 - 1·28)	0·698
Low-mod SES	1·26 (0·95 - 1·67)	0·106
Mod-high SES	1·50 (1·15 - 1·97)	0·003
Infant feeding		
Duration exclusively breast fed in months	0·98 (0·93 - 1·03)	0·339
**Lower respiratory tract infection (LRTI)**		
	Odds Ratio (95% CI)	P value
Site		
Mbekweni	1·19 (0·90 - 1·58)	0·222
Combined		
Smoke exposure	1·39 (0·98 - 1·96)	0·067
Infant characteristics		
Male	1·74 (1·34 - 2·26)	<0.001
WAZ at birth	0·94 (0·84 - 1·06)	0·304
Maternal HIV exposure	1·22 (0·83 - 1·80)	0·310
Age of EPI in months	0·90 (0·88 - 0·93)	<0.001
SES quartiles		
Lowest SES	1·20 (0·82 - 1·75)	0·348
Low-mod SES	1·58 (1·10 - 2·26)	0·013
Mod-high SES	0·95 (0·65 - 1·39)	0·796
Infant feeding		
Duration exclusively breast fed in months	0·96 (0·91 - 1·03)	0·341

WAZ, weight-for-age z-score; HIV, human immunodeficiency virus; EPI, extended programme for immunisation; SES, socio-economic status

Supplemental Table 9: Multivariate analysis for combined environmental tobacco smoke (ETS) and indoor air pollution (IAP) exposures

**Wheeze**		
	IRR (95% CI)	P value
Combined		
IAP / ETS exposure	1·96 (1·32 - 2·92)	0·001
Infant characteristics		
Male	1·42 (1·17 - 1·73)	<0·001
WAZ at birth	0·95 (0·87 - 1·03)	0·234
Maternal HIV exposure	0·51 (0·34 - 0·75)	0·001
SES quartiles		
Lowest SES	0·99 (0·73 - 1·34)	0·955
Low-mod SES	1·26 (0·96 - 1·67)	0·100
Mod-high SES	1·53 (1·17 - 2·00)	0.002
Infant feeding		
Duration exclusively breast fed in months	1·00 (0·93 - 1·03)	0·365
**Lower respiratory tract infection (LRTI)**		
	Odds Ratio (95% CI)	P value
Site		
Mbekweni	1·11 (0·84 - 1·46)	0·454
Combined		
IAP / ETS exposure	0·99 (0·66 - 1·50)	0·994
Infant characteristics		
Male	1·67 (1·30 - 2·16)	0.000
WAZ at birth	0·90 (0·83 - 1·05)	0·250
Maternal HIV exposure	1·27 (0·87 - 1·87)	0·218
Age of EPI in months	0·90 (0·88 - 0·93)	0.000
SES quartiles		
Lowest SES	1·29 (0·89 - 1·88)	0·185
Low-mod SES	1·64 (1·15 - 2·34)	0·006
Mod-high SES	0·99 (0·68 - 1·45)	0·976
Infant feeding		
Duration exclusively breast fed in months	0·97 (0·91 - 1·03)	0·311

WAZ, weight-for-age z-score; HIV, human immunodeficiency virus; EPI, extended programme for immunisation; SES, socio-economic status

References

1. Stein DJ, Seedat S, Herman AA, Heeringa SG, Moomal H, Myer L, Suliman S, Koza L, Williams D. Findings from the first South African Stress and Health Study 2007.
2. Alkire S, Foster J. Understandings and misunderstandings of multidimensional poverty measurement. *J Econ Inequal* 2011; 9: 289-314.
3. Alkire S, Foster J. Counting and multidimensional poverty measurement. *J Public Econ* 2011; 95: 476-487.
4. Vanker A, Barnett W, Nduru PM, Gie RP, Sly PD, Zar HJ. Home environment and indoor air pollution exposure in an African birth cohort study. *Science of the Total Environment* 2015; 536: 362-367.
5. Government Gazette Republic of South Africa. National Ambient Air Quality Standards. 2009 [cited 2014 August]. Available from: https://www.environment.gov.za/sites/default/files/legislations/nemaqa_airquality_g32816gon1210.pdf.
6. Vanker A, Barnett W, Brittain K, Gie RP, Koen N, Myers B, Stein DJ, Zar HJ. Antenatal and early life tobacco smoke exposure in an African birth cohort study. *Int J Tuberc Lung Dis* 2016; 20: 729-737.
7. Siemens. Immulite 1000 Nicotine Metabolite [pamphlet]. 2009 25-08-2009 [cited 2014 November]. Available from: http://www.healthcare.siemens.com/immunoassay/systems/immulite-1000-im.
8. World Health Organisation. Integrated management of childhood illness: distance learning course. . Geneva; 2014.
9. Zar HJ, Barnett W, Stadler A, Gardner-Lubbe S, Myer L, Nicol MP. Aetiology of childhood pneumonia in a well vaccinated South African birth cohort: a nested case-control study of the Drakenstein Child Health Study. *The Lancet Respiratory medicine* 2016; 4: 463-472.
10. le Roux DM, Myer L, Nicol MP, Zar HJ. Incidence of childhood pneumonia: facility-based surveillance estimate compared to measured incidence in a South African birth cohort study. *BMJ Open* 2015; 5: e009111.
11. Zar HJ, Barnett W, Myer L, Stein DJ, Nicol MP. Investigating the early-life determinants of illness in Africa: the Drakenstein Child Health Study. *Thorax* 2015; 70: 592-594.
12. Fenton TR, Nasser R, Eliasziw M, Kim JH, Bilan D, Sauve R. Validating the weight gain of preterm infants between the reference growth curve of the fetus and the term infant. *BMC pediatrics* 2013; 13: 92.
